# The Epidemiological Scenario of Human Leptospirosis in Brazil from 2015 to 2024

**DOI:** 10.3390/epidemiologia7040114

**Published:** 2026-08-20

**Authors:** George S. C. Fernandes, Bruna O. P. Azevedo, Deborah K. Damiano, Mikael V. R. Lima, Pablo P. Macena, Aline F. Teixeira, Giovana C. Barazzone, Ana L. T. O. Nascimento, Alexandre P. Y. Lopes

**Affiliations:** 1 Laboratório de Desenvolvimento de Vacinas, Instituto Butantan, 1500 Av. Vital Brasil, São Paulo 05503-900, SP, Brazil; george.fernandes@usp.br (G.S.C.F.); brunapigatto.bp@gmail.com (B.O.P.A.); deborah.damiano@usp.br (D.K.D.); mikaelrodrigues1807@gmail.com (M.V.R.L.); pablo.macena@icb.usp.br (P.P.M.); aline.rteixeira@butantan.gov.br (A.F.T.); giovana.barazzone@butantan.gov.br (G.C.B.); ana.nascimento@butantan.gov.br (A.L.T.O.N.); 2 Programa de Pós-Graduação Interunidades em Biotecnologia, Instituto de Ciências Biomédicas, Universidade de São Paulo, São Paulo 05508-900, SP, Brazil

**Keywords:** human leptospirosis, zoonosis, Brazil

## Abstract

Background: Leptospirosis is a neglected tropical disease with substantial public health impact in Brazil, closely associated with socio-environmental vulnerabilities and climatic extremes. This study analyzed the epidemiological profile and spatiotemporal distribution of leptospirosis incidence and lethality rates in Brazil from 2015 to 2024. Methods: An ecological time-series study was conducted using secondary data from the Notifiable Diseases Information System (SINAN). Variables included geographic region, probable infection environment, and occupational and educational level (ISCED-2011). Results: A total of 31,397 cases were notified, with an annual average of 3140 cases. The South and North regions exhibited the highest incidence rates, while the Northeast and Southeast presented lethality rates above the national average (9.20%). A marked reduction in notifications occurred during the COVID-19 pandemic. Infections occurred predominantly in the domiciliary environment (64%). Rural workers (27.45%) and civil construction workers (18.63%) were the most affected occupational groups, with a higher incidence rate among illiterate and low-education populations. Conclusions: The dynamics of leptospirosis in Brazil are complex and multifactorial, influenced by climatic variations and driven by deficits in basic sanitation and education level. Mitigating the disease burden requires sustained, region-specific public health strategies, targeted infrastructure improvements, and enhanced epidemiological surveillance to address underreporting.

## 1. Introduction

Leptospirosis is a zoonotic infection caused by pathogenic species of the genus *Leptospira*, with worldwide distribution and endemic occurrence in both urban and rural areas of developing countries [1]. Global estimates suggest that this disease causes more than one million severe infections and claims approximately 60,000 lives each year [2]. Based on that, the disease accounts for an estimated 2.90 million disability-adjusted life years lost annually (UI: 1.25–4.54 million), where males bear the vast majority of this burden, accounting for roughly 80% of the total impact [3]. The prevalence is substantially higher in tropical and subtropical regions than in temperate areas, largely due to the prolonged survival of leptospires in warm, humid environments. Disease outbreaks are commonly associated with the rainy season, particularly during periods of heavy rainfall and flooding [4]. In Brazil, in addition to heavy rainfall, the disease is also associated with densely populated urban regions [5].

In urban settings, rodents serve as the primary reservoir of *Leptospira*, as they typically remain asymptomatic while maintaining long-term renal colonization. Infected rodents continuously shed viable leptospires in their urine, contaminating the environment and facilitating transmission through direct contact or indirectly via contaminated soil and water [6,7,8]. Following exposure through abraded or water-sodden skin or mucous membranes, pathogenic leptospires rapidly penetrate host tissues, overcoming biological barriers and resisting complement-mediated killing. They can disseminate hematogenously and reach target organs, including the kidneys (via the proximal tubules), liver, and lungs, within one hour of infection [8], highlighting their marked invasive capacity [7].

In addition to its public health relevance, leptospirosis imposes a significant economic burden on the agricultural sector, as infection in livestock can result in abortions, stillbirths, infertility, reduced milk production, and death [8].

Prophylactic measures remain the most effective strategy for controlling leptospirosis, particularly because improvements in sanitation and sustained rodent control are often difficult to implement. Despite extensive research efforts worldwide, a universal vaccine has not yet been achieved. Currently, bacterins, formulated from inactivated whole-cell preparations, are the only commercially available vaccines. These are primarily used in veterinary medicine and, in some countries, are administered to high-risk occupational groups in humans [9,10,11]. However, bacterin-based vaccines have important limitations, including serovar-specific protection and short duration of immunity, necessitating annual booster doses.

Considering the clinical significance of leptospirosis, in recent years, several studies have been conducted to understand the epidemiology of this disease in Brazil. However, due to the complexity of the disease and lack of information, many gaps remain. Indeed, as an illness deeply intertwined with poverty, human leptospirosis remains doubly neglected due to a combination of clinical misdiagnoses and demographic marginalization, creating a pattern of systemic invisibility that disproportionately affects vulnerable populations. To address this, the present study aims to update the epidemiological profile of leptospirosis in Brazil by analyzing spatiotemporal distribution, incidence, and lethality rates from 2015 to 2024, considering available data regarding socio-environmental vulnerabilities such as the probable environment of infection, occupational sector, and educational level on the disease dynamics. These findings are expected to provide relevant insights into the epidemiology of the infection and its associated risk factors, which could support the implementation of targeted public health interventions.

## 2. Materials and Methods

### 2.1. Study Area

**Figure 1:**
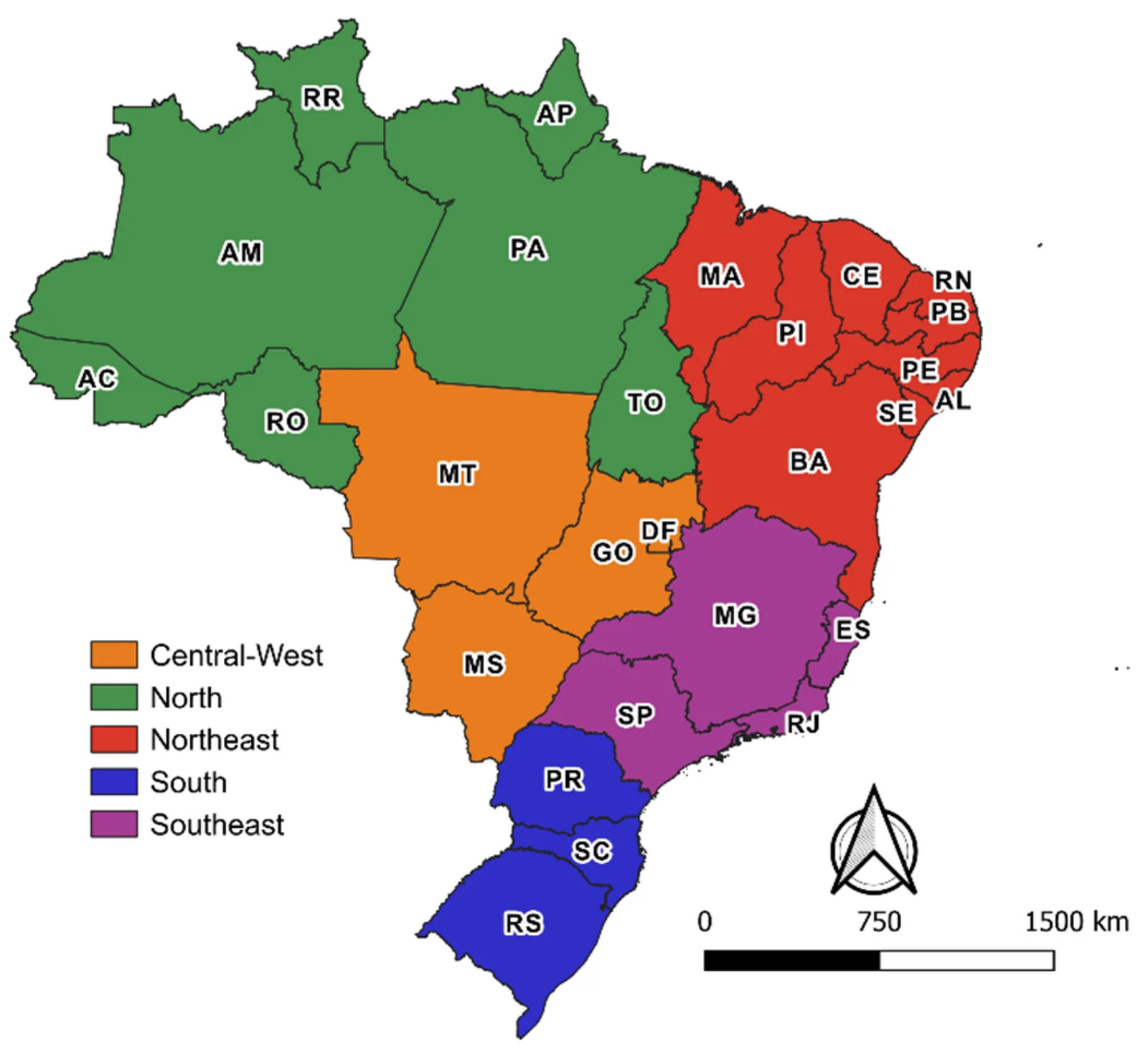
Political map of Brazil stratified by region. The map illustrates the study area and highlights the grouping of the 27 Federative Units. The colors indicate the five official regions (North, Northeast, Central–West, Southeast, and South), which served as a spatial scale for aggregating epidemiological data. AC: Acre, AL: Alagoas, AM: Amazonas, AP: Amapá, BA: Bahia, CE: Ceará, DF: Distrito Federal, ES: Espírito Santo, GO: Goiás, MA: Maranhão, MG: Minas Gerais, MS: Mato Grosso do Sul, MT: Mato Grosso, PA: Pará, PB: Paraíba, PE: Pernambuco, PI: Piauí, PR: Paraná, RJ: Rio de Janeiro, RN: Rio Grande do Norte, RO: Rondônia, RR: Roraima, RS: Rio Grande do Sul, SC: Santa Catarina, SE: Sergipe, SP: São Paulo, TO: Tocantins.

An ecological time-series study was conducted using an exploratory approach, encompassing the Brazilian national territory. The investigation period comprised the years from 2015 to 2024. The geographic analysis unit consisted of the 27 Federative Units (26 states and the Federal District), evaluated both individually and grouped into the five major regions (North, Northeast, Central–West, Southeast, and South) (Figure 1).

### 2.2. Participants (Study Population)

The study population comprised all confirmed leptospirosis cases reported in Brazil from 2015 to 2024. Secondary data were extracted from the Brazilian Notifiable Diseases Information System (SINAN), publicly available through the Informatics Department of the Unified Health System (DATASUS) (accessed on 22 September 2025). Data for 2024, updated by the Ministry of Health on 9 July 2025, are preliminary and subject to revision. To calculate incidence rates, annual resident population estimates provided by the Brazilian Institute of Geography and Statistics (IBGE) were used as denominators.

No sample size calculation was performed, as the study considered the census universe of the notifications registered in the official system (intentional non-probabilistic sampling).

### 2.3. Data Collection (Quantitative/Categorical Variables)

The variables of interest were extracted from the SINAN Notification/Investigation Form. Stratification variables included the year of notification, Federative Unit of notification, geographic region, area of residence (urban or rural), exposure setting (leisure, domiciliary, or occupational), and educational level. Based on the extracted data, the indicators analyzed were the incidence and lethality rates of leptospirosis. Because it is a retrospective study based on secondary data in the public domain, strictly anonymous and without the possibility of individual identification, the present work is exempt from review by a Research Ethics Committee, in accordance with the guidelines of the National Health Council (CNS) and the Brazilian General Data Protection Law (LGPD—Law No. 13.709/2018).

To characterize the occupational profile of the notified cases, an empirical cutoff was established by selecting the top 100 most frequent occupations reported in the Epidemiological Surveillance Panel of the Brazilian Ministry of Health. The rationale for selecting this specific threshold is based on the cumulative frequency distribution curve of the dataset. This set accounted for 83.18% of the records with the “occupation” variable filled in. Due to the severe “long-tail” dispersion inherent to occupational registries in the SINAN database, expanding the extraction beyond the top 100 yielded diminishing analytical returns. Occupations below this rank exhibited negligible individual frequencies representing statistical noise rather than consistent epidemiological patterns. In order to systematize the analysis and facilitate pattern identification, the 100 selected occupations were regrouped into eight categories defined by the nature of the economic activity and the similarity of the work processes. The remaining occupations, due to their high dispersion, were aggregated into “others and unclassified”. It should be noted that the relative frequencies presented refer exclusively to cases in which the occupation was filled out in the system of origin. Thus, the percentages presented reflect the distribution among the cases with known occupation, and the possibility of information bias resulting from the non-completion of this variable in the notification forms (ignored/blank) must be considered.

The variable regarding education, originally registered in SINAN according to the Brazilian educational system, was recoded. To ensure international analytical comparability, methodological equivalence was established in accordance with the Operational Manual of the International Standard Classification of Education (ISCED) (15). Consequently, the original strata were aggregated as follows:

- ISCED 0: illiteracy or no formal education;
- ISCED 1–2: primary education, corresponding to the 1st to 9th grade, locally termed *ensino fundamental*, and incomplete high school;
- ISCED 3–4: complete high school, referring to the 1st to 3rd year, or *ensino médio*, and incomplete higher education;
- ISCED 5–6: complete technical level or higher education, locally termed *graduação superior*.

The underlying data for the figures and tables are provided online on Zenodo (https://doi.org/10.5281/zenodo.20801665) (accessed on 22 September 2025).

### 2.4. Map Preparation

The analysis of the spatial distribution of leptospirosis in the Brazilian territory was represented by choropleth maps. For the definition of the class intervals, a partitioning approach using medians applied to the global dataset (*n* = 270 observations) was adopted, corresponding to the annual incidence rates of all 27 Federative Units over the analyzed decade. The values were arranged in ascending order: the first median divided the total set, isolating the upper half of the data; then, the median of the remaining lower subset was successively calculated, repeating the process to define the limits of the lower-incidence classes. This non-parametric stratification is justified because it allows progressive isolation of extreme values (outliers) in the upper classes, preventing atypical epidemic peaks from distorting the visual representation of the historical series and ensuring a reliable visualization of the country’s basal endemicity scenario. The territorial meshes used in the cartographic preparation were obtained from public-domain geospatial databases of the Brazilian Institute of Geography and Statistics (IBGE). Spatial data processing, attribute table pairing, and final map preparation were conducted using the QGIS 3.44.6 software.

## 3. Results

### 3.1. Overview of Leptospirosis Cases in Brazil over the 10-Year Period

Human leptospirosis is a disease that affects all Brazilian states. According to the data collected from 2015 to 2024, about 31,397 cases were notified. The number of annual cases in the country averaged 3140 (Table 1). Within this period, 2021 had the lowest number of registered cases (1744), while 2015 had the highest, with 4337 notifications (Table 2). Over these 10 years, the Central–West region was the least affected by the disease, with an average of 64 annual cases and lower variation in cases among the years.

The North region presented an average of 494 annual cases, while the Northeast had 522 registered cases. These numbers, although close, diverge strongly due to the regions’ demographic density: the North region has 4.81 inhabitants per km^2^, while the Northeast has 36.85 inhabitants per km^2^ (Table 1). The most populous regions of the country, the Southeast and the South, harbor most of the cases, with 919 and 1141, respectively.

When evaluating incidence rate, which translates into epidemiological risk, the north region follows the south region, which has the second-highest population density in the country. The highest incidence rate of the disease was in the state of Acre, with about 28.03 cases/100,000 Inh, a number higher than in any other Brazilian state. Rio Grande do Sul ranked second, followed by Santa Catarina and Paraná states. The lowest incidence rate was observed in the state of Piauí in the Northeast, with about 0.23 cases/100,000 Inh. In contrast, the state of Pernambuco presented a high index (2.30 cases/100,000 Inh), compared to the other states in the region. The Southeast presents an overall incidence rate of 1.04 cases/100,000 Inh, with higher prevalence in São Paulo and Rio de Janeiro (Table 1).

The lethality rate, a direct indicator of the clinical severity and disease outcome, was 9.20% in Brazil during the study period. However, a regional variation was observed, with the Northeast and Southeast regions standing out for presenting lethality rates above the national average. In the overall average, the Northeast registered a lethality of 14.12%, with Sergipe, Maranhão, Bahia, Pernambuco, and Rio Grande do Norte standing out for having lethality above the average. The lethality rate in Sergipe is notably high (21.34%), particularly when compared with data from Pernambuco, which has a higher incidence rate (Table 1). The Southeast region had an average lethality of 12.79%, with São Paulo and Rio de Janeiro presenting the highest lethality rates. The Central–West, North, and South regions registered the lowest indices, at 5.5 and 8%. Although the Central–West region has a lower lethality than other regions, the state of Mato Grosso and the Federal District stand out for their low incidence and lethality rates above the overall average. In contrast, in the North region, the state of Acre stands out for its very high incidence rate and a lethality rate of 0.67% (Table 1).

### 3.2. Distribution of Leptospirosis Cases by Region of Brazil

**Figure 2:**
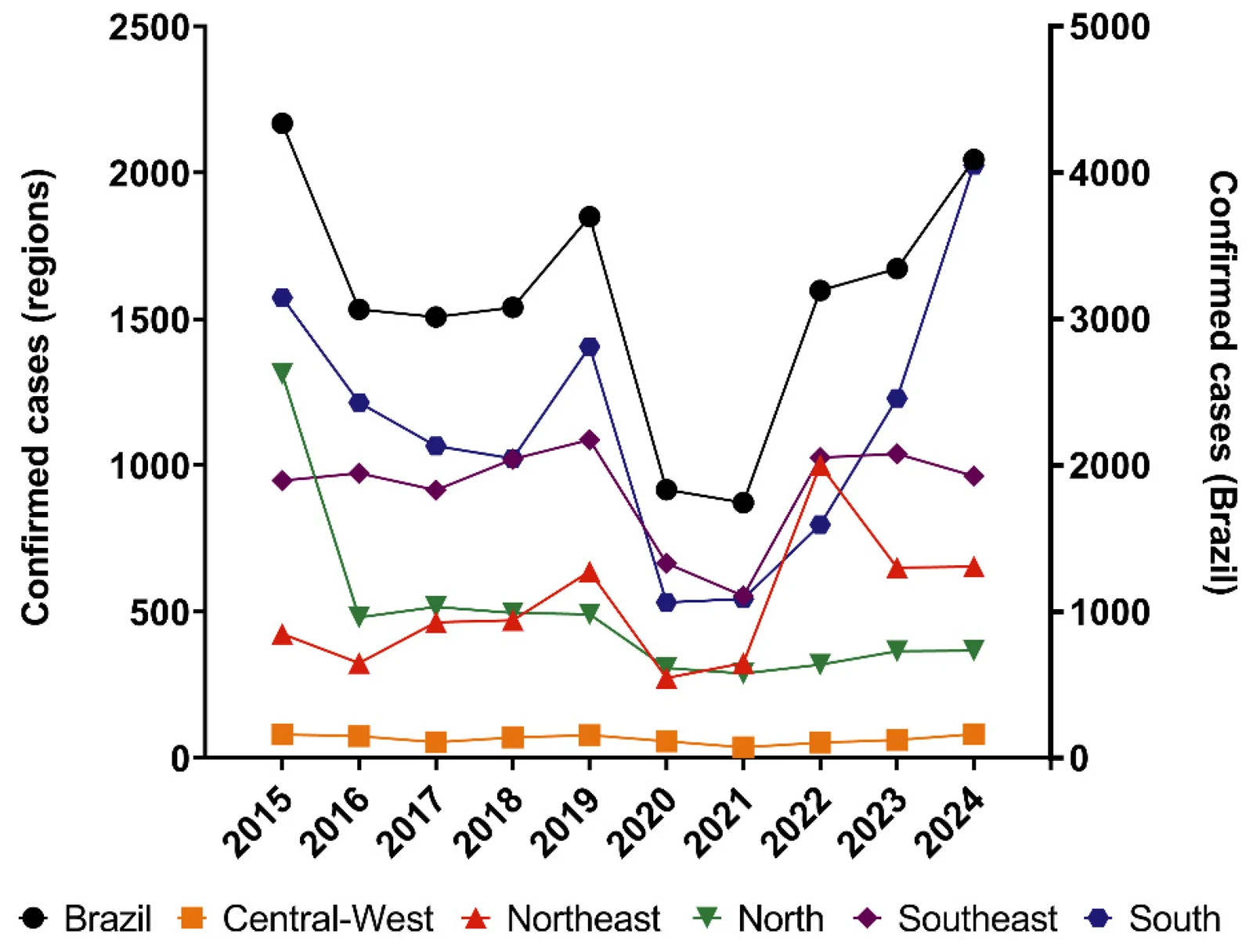
Historical series of confirmed leptospirosis cases in Brazil and regions. The left vertical axis shows the annual number of cases for each region of the country, while the right vertical axis shows the consolidated total of cases across the national territory (black line).

According to the distribution of cases over the 10 years comprised in the study, the South region began the last decade with 1573 cases in the year 2015 and followed a downward trajectory until 2018, when it reached 1023 cases, accompanying the national average, 4337 in 2015 and 3079 in 2018 (Figure 2 and Table 2). From 2022 onward, a sharp increase in cases was observed, peaking in 2024 with 2025 cases, the highest number on record. This surge was exacerbated by severe flooding in the state of Rio Grande do Sul. In the Southeast region, 948 cases were reported in 2015, followed by a gradual upward trajectory that reached a peak of 1087 cases in 2019, representing a 15% increase compared to the region’s annual average of 919 cases.

In the year 2015, the North region presented 1312 cases, far above its annual average of 494 cases, due to the numbers of the state of Acre, with an exceptional 601 cases, when the state faced an environmental disaster, with a flood considered the largest in history (the Acre river registered a record on 4 March, affecting more than 100,000 people) (Figure 2 and Table 2). From 2016 onwards, the number of cases remained close to the annual average (480 cases), with minimal variation among the other periods. In 2020 and 2021, a reduction in notifications was observed, but not as pronounced as in the south and southeast regions. In 2024, the year of the second-largest flood in the state of Acre, no significant increase in notifications was observed, ending the analyzed series with 367 notified cases. The Central–West region presented the lowest notification index during the entire analyzed period, with a reduction in the number of cases being observed only in 2021 (Table 2).

The Northeast region began the period with 424 cases in 2015. A reduction in the number of cases was observed in 2016, and from the following year onwards, reaching 637 cases, above the region’s annual average of 522 cases. Notably, an isolated peak was observed in 2022 due to catastrophic rains in several states of the region. In this period, 1000 cases were identified, momentarily deviating from the stability observed in previous and subsequent years. A reduction was observed, with 650 cases in 2023 and 654 cases in 2024 (Table 2).

Generally, in the studied regions, in the 2020–2021 biennium, a “U”-shaped pattern of descent and ascent could be identified. The generalized decline coincides with the critical period of the COVID-19 pandemic, suggesting a possible influence of underreporting or social isolation measures on the transmission dynamics. From 2022 onwards, the number of cases returned to the indices observed in the previous period.

### 3.3. Annual Incidence Rate of Leptospirosis Cases in Brazil

**Figure 3:**
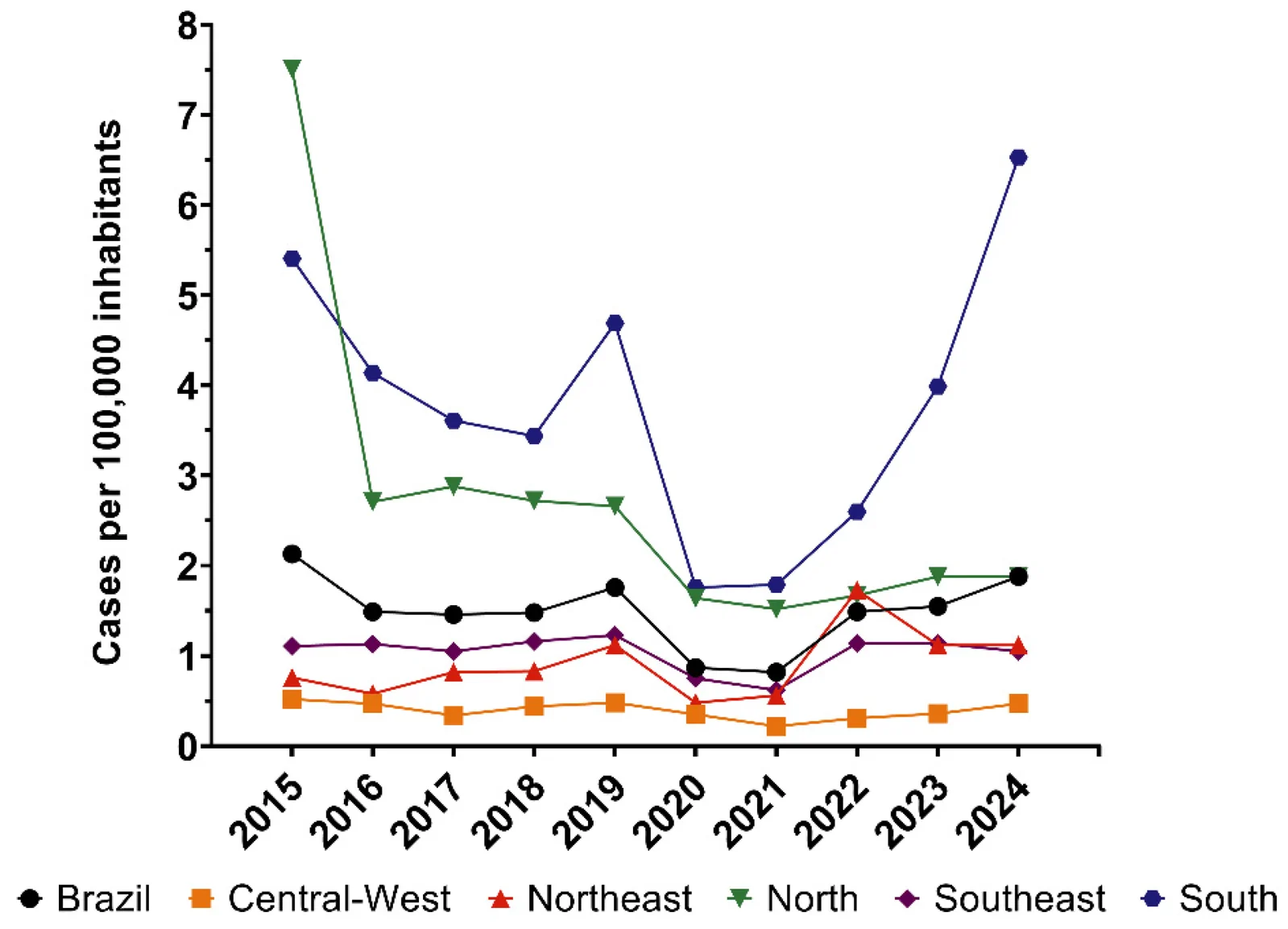
Leptospirosis incidence rate in Brazil and regions. The vertical axis indicates the annual proportion of cases per 100,000 inhabitants stratified by region and for the country (black line).

The yearly variations in incidence rates reflect the changes in the number of cases (Figure 3). It is observed that the North region leads the incidence rate in 2015 (7.51 cases/100,000 Inh), the highest recorded during the 10 years analyzed, followed by a sharp drop in subsequent years (Table 3). The South of the country, in turn, stood out for presenting the highest incidence rates throughout the evaluated decade, with expressive growth, especially between the years 2021 (1.79 cases/100,000 Inh) and 2024 (6.53 cases/100,000 Inh). The Southeast and Northeast Regions maintained incidence rates below the annual national average (1.49 cases/100,000 Inh) during most of the analyzed period. However, that the Northeast Region presented a significant incidence rate peak in 2022 (1.73 cases/100,000 Inh). The Central–West region stands out for having a lower incidence rate than the other regions, and the maximum observed incidence rate was 0.52 cases/100,000 Inh in 2015 (Table 3).

### 3.4. Distribution of Leptospirosis Incidence Rates by States of Brazil

**Figure 4:**
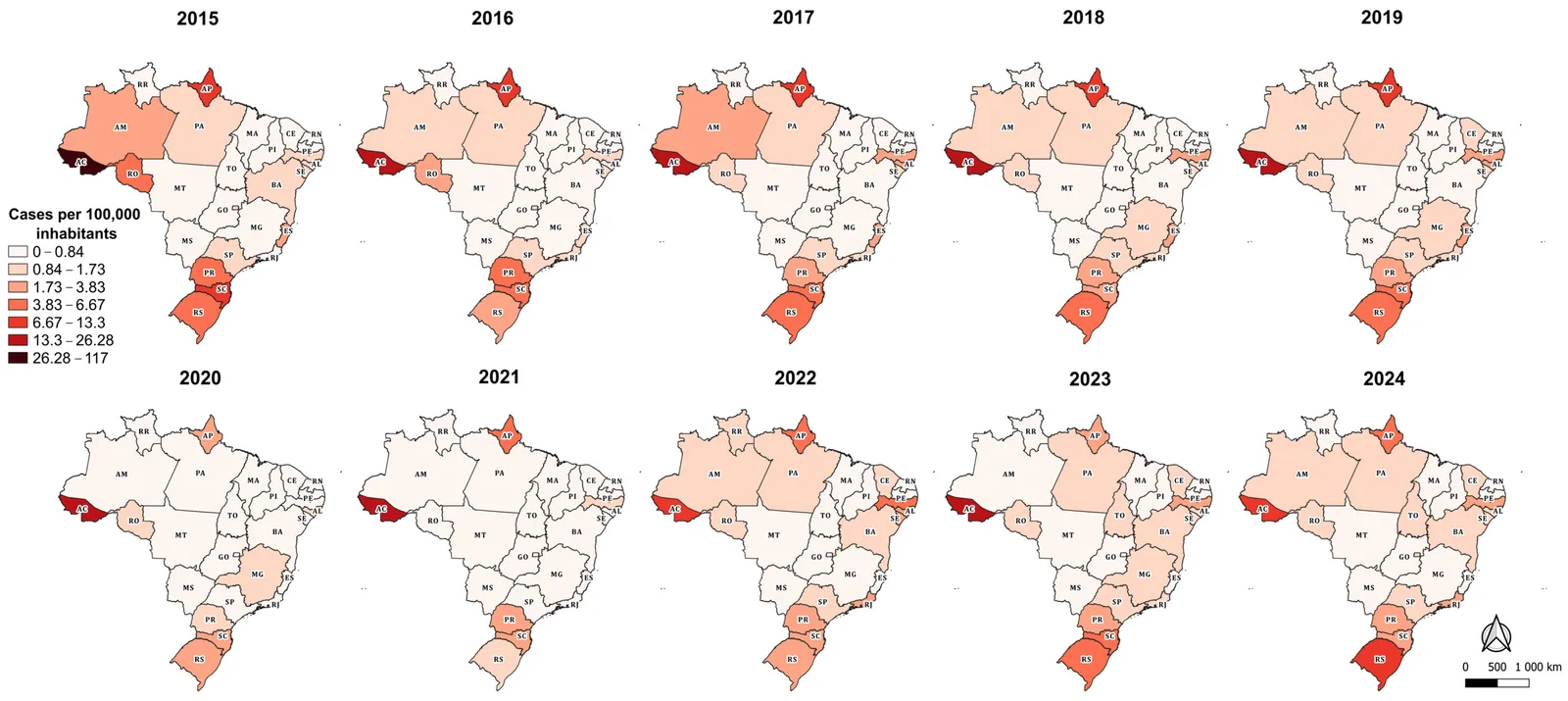
Spatiotemporal distribution of leptospirosis incidence rates in Brazil by federative unit (2015–2024). The series of annual choropleth maps illustrates the incidence rate (cases per 100,000 inhabitants). To ensure visual comparability throughout the decade and to attenuate the visual flattening caused by high-incidence rate outliers (e.g., the state of Acre), the continuous color scale was stratified into six classes using the recursive median division method applied to the total dataset for the period. Darker shades of red indicate the highest registered incidence rates. AC: Acre, AL: Alagoas, AM: Amazonas, AP: Amapá, BA: Bahia, CE: Ceará, ES: Espírito Santo, GO: Goiás, MA: Maranhão, MG: Minas Gerais, MS: Mato Grosso do Sul, MT: Mato Grosso, PA: Pará, PE: Pernambuco, PI: Piauí, PR: Paraná, RJ: Rio de Janeiro, RN: Rio Grande do Norte, RO: Rondônia, RR: Roraima, RS: Rio Grande do Sul, SC: Santa Catarina, SE: Sergipe, SP: São Paulo, TO: Tocantins.

Although the northern and southern regions have the highest incidence rates, the disease’s dynamics are complex. The analysis of the historical series of the geographic distribution of leptospirosis incidence rates in Brazil corroborates the complexity of this scenario (Figure 4). A persistently high disease burden is observed in states such as Acre and Amapá, which characterizes leptospirosis as endemic in these locations. The South region, in turn, exhibits consistently high incidence rates with periods of exacerbation that constitute epidemic outbreaks. Some states, such as Pernambuco and Espírito Santo, present a pattern of lower magnitude, marked by sporadic epidemic episodes. Additionally, the states Bahia, Tocantins, and Ceará had an increase in incidence rates from 2022 onwards, in comparison to the previous period, which differed from the “U”-shaped pattern described above.

### 3.5. Lethality Index of Leptospirosis in Brazil

**Figure 5:**
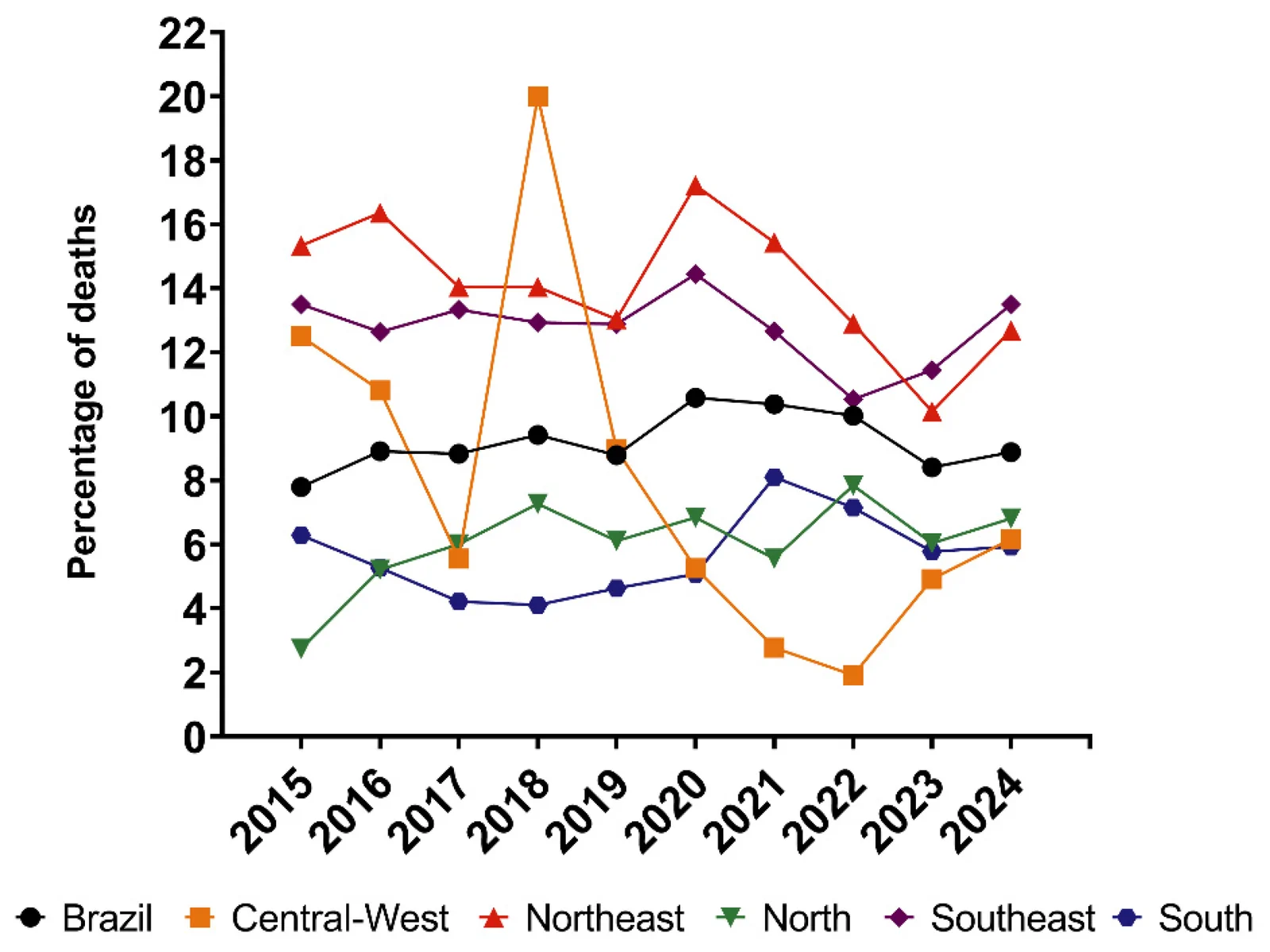
Leptospirosis lethality rate in Brazil and regions. The vertical axis shows the annual percentage of deaths relative to the total confirmed cases, stratified by region and for the national consolidated (black line).

The average lethality of leptospirosis in Brazil remained basically constant over the 10 years, varying from 7.79% to 10.58% of cases that evolved to death (Figure 5 and Table 4). The Northeast and Southeast regions had lethality indices above the national average, reaching 17.22 and 14.44, respectively. The regions with the highest incidence rates of the disease, the South and North, remained below the national average, with minimums of 4.11 in 2018 and 2.74 in 2015, respectively. The Central–West region presents a graphically large variation due to the low number of cases, which consequently is highly impacted by a low number of deaths.

### 3.6. Distribution of Leptospirosis Cases According to Environment and Occupation

**Figure 6:**
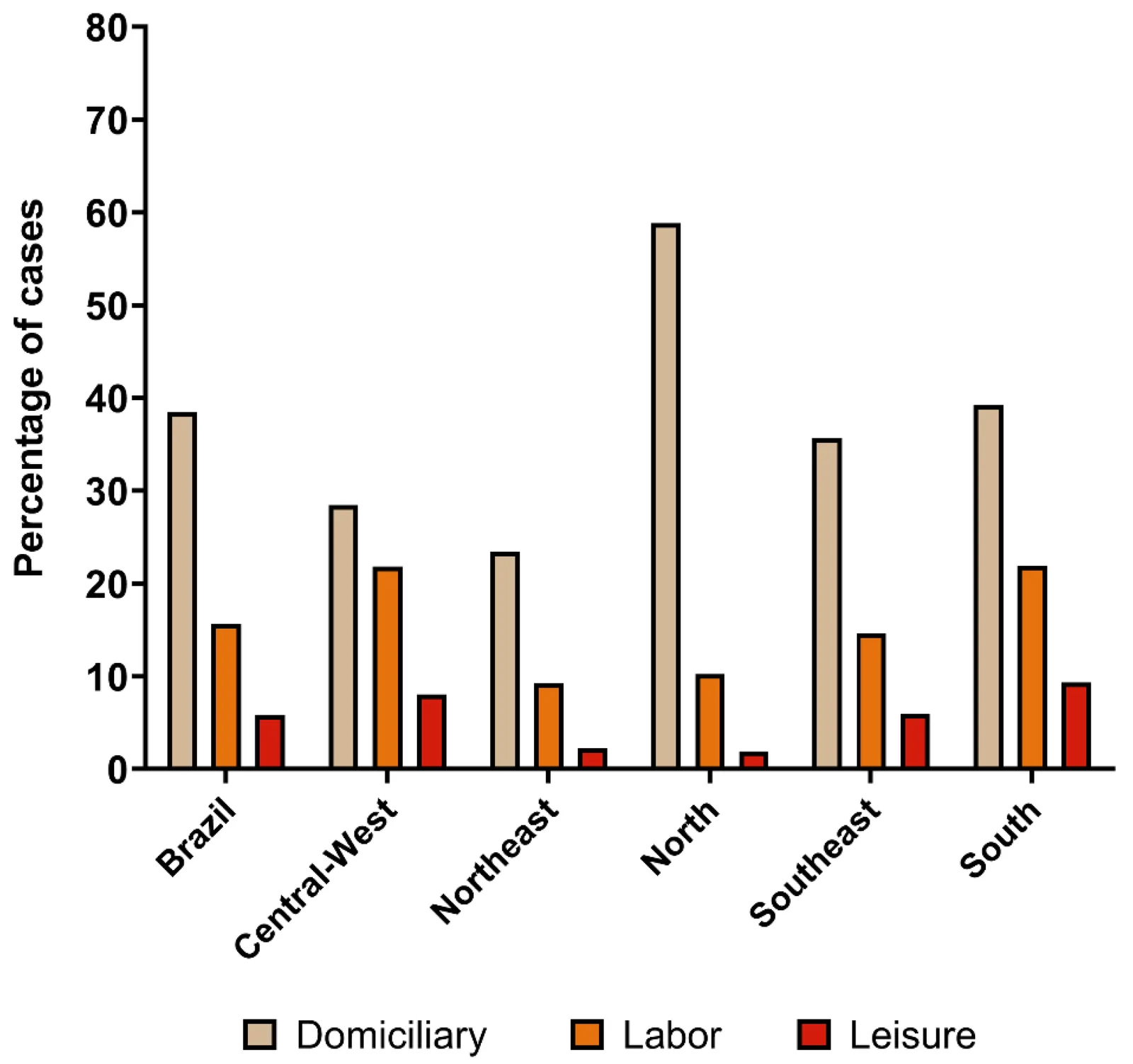
Proportion of leptospirosis cases according to the probable environment of infection in Brazil and regions (2015–2024). The vertical axis indicates the percentage of notifications in domiciliary, work, and leisure environments. The calculation exclusively considered the records with a valid location of infection, excluding blank or ignored data. The “other environments” category includes the percentage calculation; however, it was omitted from the graph due to the unspecificity of this variable in the registration forms.

Among the 31,397 notified cases over the period, only 18,894 cases, corresponding to 60.1% of the total, reported the type of environment where the contamination occurred. Of the contaminations with indicated locations in Brazil, 64% occurred in residential environments, 26% in workplaces, and 10% during leisure activities (Figure 6 and Table 5). The distribution pattern found in Brazil is repeated across all regions. In the North region, the number of domiciliary cases clearly exceeds the annual average, at 82%. Cases linked to the occupational environment are higher in the Central–West and South compared to the other regions, at 38% and 30%, respectively; however, the Central–West has a small sample size, and therefore, the percentages may be unrepresentative. The cases related to leisure range from 13% in the Central–West and South regions to 3% in the North region.

**Figure 7:**
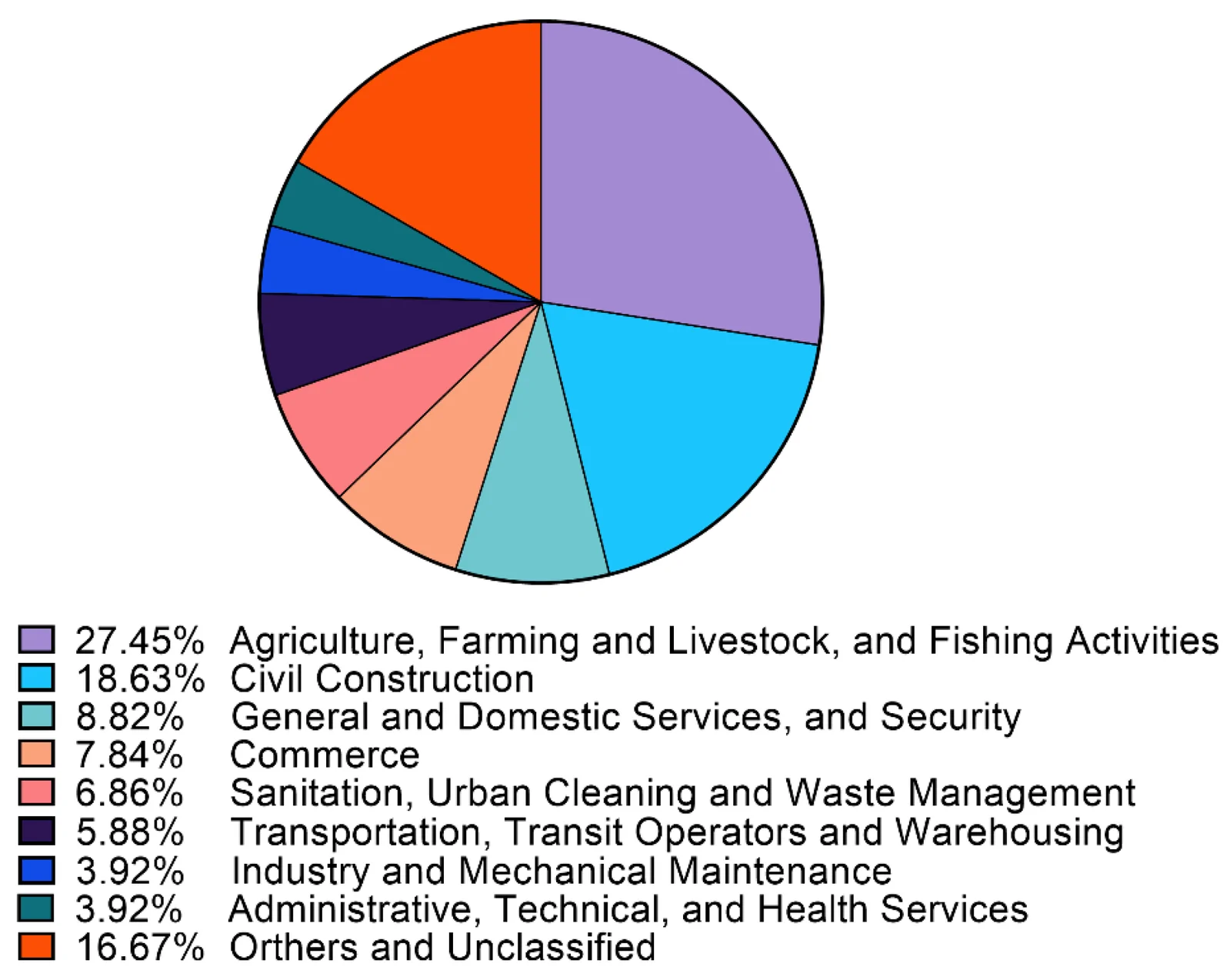
Percentage distribution of leptospirosis cases according to occupation (2015–2024). The illustrated categories represent the grouping of the 100 most frequent occupations registered in the Epidemiological Surveillance Panel, corresponding to 83.33% of the total notifications in the period. The “Others and unclassified” category (16.67%) encompasses the residual volume of occupations not listed in the main ranking or not specified.

According to the epidemiological surveillance panel, among the leptospirosis notifications that informed the occupation, it was identified that rural workers in agriculture, livestock farming, fishing, and other related activities accounted for 27.45% of the cases. Subsequently, jobs related to civil construction in general account for 18.63% of the cases. It is interesting to note that these two types of occupation, when added together, represent a significant portion of the cases with known occupation, accounting for 46.08%. Occupations related to industry, administrative, and health services account for the lowest percentage of cases, with about 3.92%. Furthermore, a significant number of notified cases (16.67%) were not related to a specific occupation. It is important to emphasize that these percentages correspond only to the number of cases with the occupation declared in the notification and, furthermore, do not necessarily reflect a relationship with the location of contamination (Figure 7).

### 3.7. Distribution of Leptospirosis Cases by Education Level

**Figure 8:**
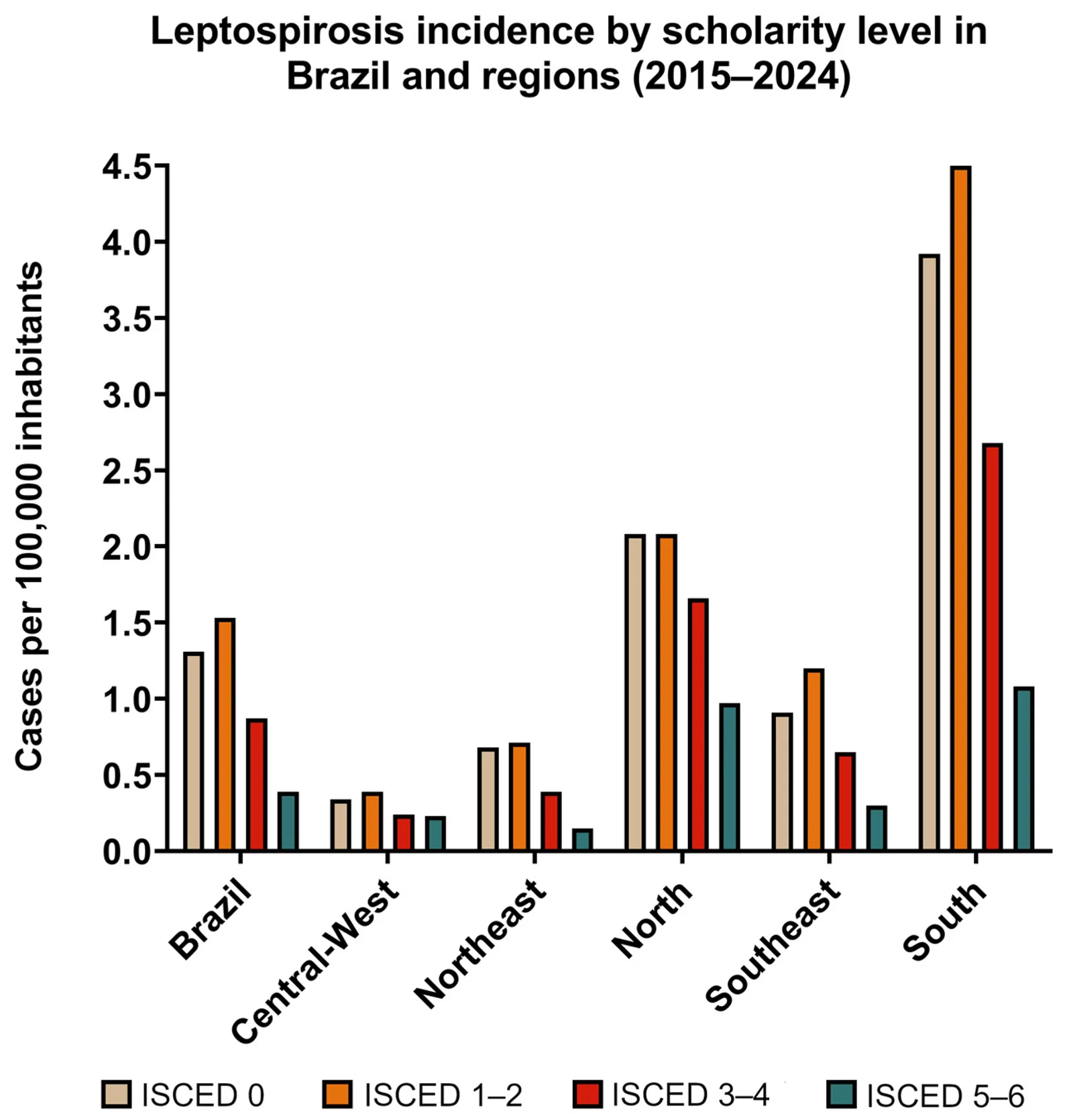
Leptospirosis incidence rate according to educational level in Brazil and regions (2015–2024). The vertical axis shows the average incidence rate (cases per 100,000 inhabitants), stratified by each educational group’s population size. The categories were standardized according to the International Standard Classification of Education (ISCED-2011): ISCED 0 (no formal education/illiteracy); ISCED 1–2 (elementary and incomplete high school); ISCED 3–4 (complete high school and incomplete higher education); and ISCED 5–6 (complete technical level or higher education).

To describe the distribution of cases according to education level, the International Standard Classification of Education (ISCED) was used. The illiterate Brazilian population, classified as ISCED 0, together with the population with low education level, ISCED 1–2, presented incidence rates of 1.31 and 1.53 cases per 100,000 inhabitants, respectively. For the population with medium and high education levels, ISCED 3–4 and 5–6, rates of 0.87 and 0.39 cases per 100,000 inhabitants were calculated, respectively. This same pattern of reduction in incidence rates with the increase in education is observed in different proportions among the Brazilian regions and states, being notably evidenced in the south region, which has an incidence rates of 3.92 and 4.5 per 100,000 inhabitants for categories 0 and 1–2, while for categories 3–4 a reduction to 2.68 per 100,000 inhabitants was observed, which becomes even more notable in categories 5–6 with 1.08 cases per 100,000 inhabitants (Figure 8 and Table 6).

## 4. Discussion

Although leptospirosis is internationally recognized as a neglected tropical disease, in Brazil, despite being considered an important public health problem, it was not included in the epidemiological bulletin of neglected diseases [12]. Therefore, there is no Brazilian funding program aimed at the intervention of this disease. However, taking into account the potential years of life lost and the hospital costs of leptospirosis in Brazil [13], this disease deserves prominence. In this study, a total of 31,397 leptospirosis cases were identified over the last 10 years, with an annual average of 3140 cases. The annual average of cases apparently did not show large variations over long periods, since a previous analysis conducted from 2000 to 2015 [14], reported an average of 3810 annual cases. This average coincided with the data analyzed from 2007 to 2017, with 3846 cases [15]. Furthermore, considering the average annual number of cases observed from 1996 to 2005 (3165 notifications), we can suggest that the number of cases in Brazil remained stable over these years [16]. Although this maintenance is already expected due to the absence of a vaccine for human use [17] and the lack of significant improvements in the sanitary conditions of the cities, we must be aware that the lack of diagnosis in the initial phase of the disease can lead to underreporting of data. The study conducted from 1996 to 2005 showed that Brazil was responsible for about 86% of the cases across South America [16], emphasizing the importance of this zoonosis to the country.

The temporal dynamics of this survey showed a reduction in notifications across all regions during the COVID-19 pandemic. This pattern was not exclusive to leptospirosis, with a decrease observed in other compulsorily notifiable diseases in Brazil [18,19] and in other countries, such as China and Australia [20,21]. However, rather than being a localized anomaly, this phenomenon reflects a well-documented worldwide disruption of public health systems [22]. The massive reallocation of laboratory capacities and personnel toward SARS-CoV-2 mitigation led to severe underreporting. Furthermore, this reduction was likely multifactorial globally: lockdowns and mobility restrictions temporarily altered environmental and occupational exposure patterns [23], while reduced healthcare-seeking behavior, driven by the fear of hospital-acquired COVID-19 infections, prevented patients with febrile illnesses from seeking early diagnosis [24]. This strongly suggests that, due to the health system’s focus on the pandemic, there was a widespread failure in notifications. This premise of underreporting is heavily reinforced by our findings regarding the maintenance of the lethality index during the period, particularly in the Southeast and Northeast. The evaluation of the epidemiological determinants of leptospirosis requires a critical reflection on the effect of geographic scale. While the present analysis at the national level offers a comprehensive panorama, studies at the municipal scale tend to provide greater precision in capturing socioenvironmental nuances, as demonstrated in a previous study in Rio de Janeiro [25]. However, extrapolating hyper-localized analyses poses limitations given Brazil’s continental dimensions. In this sense, the formulation of a regional and systemic vision is indispensable.

The analysis of the location of contamination reveals that the domiciliary environment represents the main route of exposure (64%), followed by the occupational environment (26%) and recreational activities (10%). This profile reflects the dynamics of low- and middle-income countries, where precarious sanitary infrastructure is a determinant of the infection [26,27], contrasting with high-income nations, where sports and leisure predominate. In Brazilian urban settings, proximity to open sewers and the accumulation of garbage promote the proliferation of synanthropic rodents (*Rattus norvegicus*), thereby consolidating the domiciliary as the epicenter of transmission among marginalized populations [28,29].

Regionally, the disparity is evident in the North, where the residential environment accounts for 82% of contaminations, indicating a critical deficit in sanitation [30]. On the other hand, the Central–West and South had higher proportions of occupational cases (38% and 30%), indicating an interface with agribusiness. From an occupational perspective, the predominance of rural (27.45%) and civil construction workers (18.63%) underlines the overlap between unsanitary work and environmental risk [31]. This vulnerability is corroborated by education level (ISCED): the highest incidence rates occur in illiterate populations (1.31/100,000) and in those with low education (1.53/100,000), consolidating the premise that education and income determine socio-spatial resilience [26,32]. The drastic decline in cases in the most educated strata evidences that favored populations inhabit zones with urban infrastructure that acts as a barrier against floods [33,34]. Furthermore, higher education commonly generates formal employment with higher remuneration; it was calculated that for every one-dollar increase in daily per capita expenditure per household, the chances of primary infection are reduced by 50% [29].

Regarding clinical outcomes, no direct relationship between incidence rate and lethality was observed across regions. In general, the North and South stand out for presenting high incidence and low lethality rates. In contrast, the largest number of cases in urban areas occurred in the Northeast [14], which had the lowest incidence rate but the highest lethality rate during the period. This scenario suggests a specific failure in the early diagnosis of the disease. The state of Sergipe draws attention due to the elevated number of deaths: A study conducted in the state pointed out that, of 516 notified cases (2007–2022), 140 evolved to death (lethality of 27.2%) [35]. Similar to previous observations [36,37], the highest prevalence occurs in men of economically active age. The interregional lethality discrepancy raises the hypothesis that areas with higher mortality, such as the Southeast, may have a greater circulation of highly virulent strains, such as those of the Icterohaemorrhagiae serogroup. However, because the national surveillance system (SINAN) relies on standardized notification forms that do not capture serovar identification, time-to-hospitalization, or specific therapeutic protocols, empirically evaluating these clinical determinants remains a structural limitation of secondary database analyses in Brazil.

A recent study by Marteli et al. (2025) [38], based on SINAN data and statistical models, showed that residing in the Southeast and Northeast increases the adjusted odds of death from leptospirosis by 83% and 40%, respectively, compared to the North. The authors linked these fatal outcomes to social health inequalities, emergency department overcrowding, delayed diagnosis, and limited access to intensive care and renal replacement therapy. The interregional lethality discrepancy, notably higher in the Northeast and Southeast, reflects complex interactions between biological determinants and healthcare infrastructure. While climatic factors and extreme rainfall primarily drive transmission incidence rate [39], severe meteorological events in areas with inadequate infrastructure disrupt healthcare logistics and overload local emergency services, indirectly contributing to lethality by delaying hospital admission. The high lethality in the Northeast, reflected in our results, suggests this interaction between inadequate sanitation infrastructure and climatic factors.

Globally, leptospirosis outbreaks are associated with extreme weather events [40]. In São Paulo, studies demonstrated a 31.5% increase in hospitalizations for every 20 mm of rain [41]. In the South, these events induce the formation of risk clusters of leptospirosis dependent on hydrological disasters [42,43], culminating in catastrophic outbreaks, such as the one in Rio Grande do Sul in 2024 [44,45].

The Amazon basin exhibits a complex epidemiological mosaic. The state of Acre draws attention due to the alarming number of persistent cases. The relationship with the climate reflects the dynamics of the Acre River, with recurrent overflow episodes. Associated factors, such as disorganized urban development along the riverbanks and increased reservoir density, including rodents and capybaras [46], favor local transmission. In 2015, Acre faced the largest flood in its history [47], which justified the extreme peak of notifications. However, in the second-worst overflow, in 2024, the average of notifications remained similar to previous years. This epidemiological plateau, allied with the state’s history of declaring environmental emergencies [48], suggests the effect of continuous, more active epidemiological surveillance in a known endemic region, whose peaks characteristically occur in March [49]. The severity of these floods demands continuous strategies, such as the Emergency Plan for Coping with Floods [50]. In parallel, Amapá also stands out due to the high endemism in the Guianas region, which actually consisted of underreporting and clinical confounding with malaria and arboviruses [51]. It is important to emphasize that the associations between incidence rate peaks and extreme weather events discussed in this study remain descriptive. Because primary meteorological and environmental variables were not incorporated into our statistical models, these temporal and spatial overlaps highlight socio-environmental vulnerabilities but should not be interpreted as formal causal relationships, even though they align with well-documented historical events and existing literature.

An important methodological aspect of this study concerns the nature of the database used. The Brazilian Information System for Notifiable Diseases (SINAN) provides strictly anonymized data, precluding longitudinal patient tracking. However, unlike administrative hospital databases, SINAN is a surveillance system that internally deduplicates records related to the same acute episode. Furthermore, human leptospirosis is an acute biphasic infectious disease that does not evolve into a chronic systemic infection in humans and elicits serovar-specific immunity upon recovery. Consequently, any rare subsequent notifications for the same individual over the years represent true reinfections, usually due to a new environmental exposure or a different Leptospira serovar, rather than a prevalent readmission of a past episode. This combination of structural database curation and the disease’s acute clinical course substantially mitigates the risk of overlapping incidence and prevalence, obviating the need for a prior washout period.

This study presents inherent limitations arising from its retrospective nature and Brazil’s vast geographic dimensions, which potentially affect secondary data from SINAN. Furthermore, the reliability of population-based epidemiological analyses is strictly conditioned by the quality and completeness of compulsory notification forms. In Brazil, passive surveillance is frequently affected by underreporting, operational inconsistencies, and a high proportion of missing or blank fields [52,53,54]. These challenges are compounded by territorial disparities in healthcare infrastructure, diagnostic availability, and the overall performance of health surveillance networks across different municipalities [55,56]. Such regional asymmetries can introduce underreporting biases and may underrepresent the true disease burden in less structured areas. Therefore, it is important to strengthen notification systems to support more precise public health policies. Furthermore, temporal changes in local municipal health system performance throughout the study period could influence notification dynamics. Nonetheless, the system’s overall importance remains noteworthy, and the presented data offer valuable insights into these findings.

As an ecological time-series study designed for macroscopic epidemiological surveillance, this investigation intentionally prioritized a descriptive and exploratory framework. While this nationwide design does not incorporate inferential statistical modeling or spatial autocorrelation algorithms to measure causal dependencies or discrete statistical clustering, it establishes a foundational baseline of leptospirosis morbidity and lethality across Brazil over a 10-year period. The observed spatial and temporal patterns documented here provide essential macroscopic insights that identify high-risk strata, thereby directing future analytical studies and supporting the rational allocation of public health resources in endemic regions.

## 5. Conclusions

The dynamics of human leptospirosis in Brazil transcend its infectious etiology, consolidating the disease as an acute marker of socioenvironmental vulnerability and infrastructural inequity. This ten-year epidemiological survey demonstrates that notifications occur predominantly in domiciliary settings, with higher rates among populations with lower educational attainment and in occupational groups exposed to unsanitary environments, particularly agricultural and construction workers. Furthermore, the spatiotemporal distribution of cases exhibits marked regional heterogeneity, coinciding with areas historically subject to severe climatic events and flooding. Consequently, mitigating the burden of this zoonosis requires an intersectoral public health approach that combines targeted improvements in basic sanitation and environmental infrastructure with sustained, region-specific epidemiological surveillance.

## Figures and Tables

**Table 1:** Average of the epidemiological indicators of leptospirosis in Brazil (2015–2024).

Study Areas	Abbr.	Demographic Density (Inh./km^2^)	Annual Mean Cases	Incidence Rate (Cases/100,000 Inh.)	Lethality (%)
Brazil	BR	24.77	3140	1.49	9.20
Central–West		10.20	64	0.39	7.89
Distrito Federal	DF	526.28	22	0.72	11.39
Goiás	GO	20.75	20	0.28	6.73
Mato Grosso	MG	3.88	15	0.41	10.84
Mato Grosso do Sul	MS	7.82	9	0.31	3.16
North		4.81	494	2.71	6.04
Acre	AC	5.41	242	28.03	0.67
Amapá	AP	6.00	52	6.20	5.20
Amazonas	AM	2.68	49	1.18	11.29
Pará	PA	6.94	111	1.28	14.43
Rondônia	RO	7.51	30	1.71	6.36
Roraima	RR	2.73	3	0.39	1.85
Tocantins	TO	5.70	8	0.49	0.71
Northeast		36.85	522	0.91	14.12
Alagoas	AL	120.13	47	1.41	13.20
Bahia	BA	26.37	96	0.65	14.60
Ceará	CE	61.48	65	0.70	12.18
Maranhão	MA	21.52	26	0.37	16.38
Paraíba	PB	71.32	18	0.43	12.50
Pernambuco	PE	97.72	222	2.30	14.56
Piauí	PI	13.01	8	0.23	0.00
Rio Grande do Norte	RN	66.62	13	0.36	14.50
Sergipe	SE	105.17	29	1.24	21.34
South		52.12	1141	3.80	5.66
Paraná	PR	57.54	349	3.05	8.09
Rio Grande do Sul	RS	40.44	508	4.45	4.92
Santa Catarina	SC	75.24	284	3.98	4.24
Southeast		95.85	919	1.04	12.79
Espírito Santo	ES	87.65	63	0.95	3.15
Minas Gerais	MG	36.17	167	0.79	7.81
Rio de Janeiro	RJ	395.48	214	1.23	15.46
São Paulo	SP	185.58	500	1.09	14.49

It presents the annual average of cases, incidence rate (per 100,000 Inh), and lethality (%) by region (highlighted), Federative Unit, and country, along with the respective population density. Abbr.: State abbreviation; Inh.: Inhabitants.

**Table 2:** Absolute number of confirmed leptospirosis cases in Brazil and regions.

Leptospirosis Cases in Brazilian Regions per Year						
Year	Brazil	Central–West	North	Northeast	South	Southeast
2015	4337	80	1312	424	1573	948
2016	3065	74	480	324	1214	973
2017	3014	54	516	463	1066	915
2018	3079	70	495	470	1023	1021
2019	3698	78	491	637	1405	1087
2020	1833	57	307	273	531	665
2021	1744	36	288	324	543	553
2022	3194	52	319	1000	797	1026
2023	3343	61	364	650	1229	1039
2024	4090	81	367	654	2025	963

The data detail the annual notifications graphically represented in Figure 2.

**Table 3:** Leptospirosis incidence rate in Brazil and by region.

Leptospirosis Incidence Rate in Brazilian Regions per Year						
Year	Brazil	Central–West	North	Northeast	South	Southeast
2015	2.13	0.52	7.51	0.76	5.41	1.11
2016	1.49	0.47	2.71	0.58	4.14	1.13
2017	1.46	0.34	2.88	0.82	3.61	1.05
2018	1.48	0.44	2.72	0.83	3.44	1.16
2019	1.76	0.48	2.66	1.12	4.69	1.23
2020	0.87	0.35	1.64	0.48	1.76	0.75
2021	0.82	0.22	1.52	0.56	1.79	0.62
2022	1.49	0.31	1.67	1.73	2.60	1.14
2023	1.55	0.36	1.88	1.12	3.99	1.14
2024	1.88	0.47	1.88	1.12	6.53	1.05

The data detail the annual proportions (cases per 100,000 inhabitants) graphically represented in Figure 3.

**Table 4:** Leptospirosis lethality rate in Brazil and by region.

Leptospirosis Lethality in Brazilian Regions per Year						
Year	Brazil	Central–West	North	Northeast	South	Southeast
2015	7.79	12.50	2.74	15.33	6.29	13.50
2016	8.91	10.81	5.21	16.36	5.27	12.64
2017	8.83	5.56	6.01	14.04	4.22	13.33
2018	9.42	20.00	7.27	14.04	4.11	12.93
2019	8.79	8.97	6.11	13.03	4.63	12.88
2020	10.58	5.26	6.84	17.22	5.08	14.44
2021	10.38	2.78	5.56	15.43	8.10	12.66
2022	10.02	1.92	7.84	12.90	7.15	10.53
2023	8.41	4.92	6.04	10.15	5.78	11.45
2024	8.88	6.17	6.81	12.69	5.93	13.50

The data detail the annual percentages of deaths graphically represented in Figure 4.

**Table 5:** Annual average of leptospirosis cases according to the probable environment of infection by region and federative unit in Brazil (2015–2024).

Subdivision	Domiciliary	Labor	Leisure
Brazil	1212	494	183
Central–West	19	14	5
Distrito Federal	6	6	3
Goiás	6	4	2
Mato Grosso	4	5	2
Mato Grosso do Sul	3	1	2
North	287	55	8
Acre	153	25	2
Amapá	39	3	3
Amazonas	24	7	2
Pará	50	14	3
Rondônia	18	5	2
Roraima	2	2	1
Tocantins	4	2	3
Northeast	126	48	11
Alagoas	14	7	3
Bahia	23	10	2
Ceará	19	7	2
Maranhão	10	3	2
Paraíba	5	3	1
Pernambuco	42	11	3
Piauí	3	2	1
Rio Grande do Norte	4	4	2
Sergipe	10	6	2
South	451	243	103
Paraná	133	77	39
Rio Grande do Sul	196	97	38
Santa Catarina	123	69	27
Southeast	329	135	55
Espírito Santo	23	14	2
Minas Gerais	57	36	12
Rio de Janeiro	84	21	6
São Paulo	178	71	36

The values represent the absolute average of the analyzed decade for notifications in domiciliary, work, and leisure environments by region (highlighted), Federative Unit, and country.

**Table 6:** Leptospirosis incidence rate according to the educational level by region and federative unit in Brazil (2015–2024).

Study Areas	ISCED 0	ISCED 1–2	ISCED 3–4	ISCED 5–6
Brazil	1.31	1.53	0.87	0.39
Central–West	0.34	0.39	0.24	0.23
Distrito Federal	0.54	0.84	0.39	0.10
Goiás	0.28	0.27	0.20	0.35
Mato Grosso	0.40	0.50	0.31	0.37
Mato Grosso do Sul	0.28	0.18	0.17	0.14
North	2.08	2.08	1.66	0.97
Acre	18.19	17.61	15.92	7.38
Amapá	6.14	5.11	2.95	2.05
Amazonas	2.27	1.56	1.53	0.07
Pará	1.07	1.86	1.36	1.03
Rondônia	1.40	1.27	0.75	0.46
Roraima	0.42	0.18	0.46	0.30
Tocantins	0.58	0.60	0.73	0.21
Northeast	0.68	0.71	0.39	0.15
Alagoas	2.32	2.61	0.82	0.26
Bahia	0.53	0.45	0.26	0.20
Ceará	2.69	2.69	1.27	0.56
Maranhão	0.36	0.61	0.29	0.21
Paraíba	0.29	0.43	0.46	0.04
Pernambuco	1.37	1.55	0.76	0.19
Piauí	0.21	0.24	0.25	0.12
Rio Grande do Norte	0.22	0.17	0.19	0.04
Sergipe	1.39	1.05	0.72	0.12
South	3.92	4.50	2.68	1.08
Paraná	2.92	4.13	2.24	0.71
Rio Grande do Sul	4.15	4.55	2.54	1.08
Santa Catarina	6.07	5.66	4.24	1.65
Southeast	0.91	1.20	0.65	0.30
Espírito Santo	0.94	1.00	0.55	0.38
Minas Gerais	0.60	0.92	0.57	0.45
Rio de Janeiro	0.80	0.91	0.41	0.17
São Paulo	2.14	2.83	1.91	0.73

The values represent the incidence rate averages (cases per 100,000 inhabitants of the respective population stratum), categorized according to the ISCED-2011 classification by region (highlighted), Federative Unit, and country, complementing the data in Figure 8 at the state level.

## Data Availability

All research data pertaining to this study are available at https://doi.org/10.5281/zenodo.20801665 (accessed on 22 September 2025) and at http://tabnet.datasus.gov.br/cgi/deftohtm.exe?sinannet/cnv/leptobr.def (SINAN) (accessed on 22 September 2025).

## Abbreviations

The following abbreviations are used in this manuscript:

SINAN	Notifiable Diseases Information System) (*Sistema de Informação de Agravos de Notificação*)
ISCED	International Standard Classification of Education
LPSs	Lipopolysaccharides
DATASUS	Department of the Unified Health System (*Departamento de Informática do Sistema Único de Saúde*)
IBGE	Brazilian Institute of Geography and Statistics (*Insituto Brasileiro de Geografia e Estatística*)
CNS	National Health Council (*Conselho Nacional de Saúde*)
LGPD	General Data Protection Law (*Lei Geral de Proteção de Dados*)
